# Supplementary material for: Protein Extraction Methods Suitable for Muscle Tissue Proteomic Analysis
Source: Proteomes. 2024 Sep 25;12(4):27. doi: 10.3390/proteomes12040027 (PMC11503273; doi:10.3390/proteomes12040027)
Supplement: Supplementary file 1 [file proteomes-12-00027-s001.zip › proteomes-3116957-supplementary.pdf]

Table S1. Proteomic data and mass spectrometry results of differential spots. Table reporting the statistically relevant spots between method A and B by spot numbers corresponding to that in figure 3 and 4, protein name, UniProt accession number, gene name, fold change, anova p-values, extraction method A and B means and ratios of %V, pI and MW and Mascot Search Results such as Score, matched peptides and Coverage %. The bold of the numbers means that number is bigger.

Spot n.	Protein name	Entry name	Gene name	AC	Anova test			Fold Change	Theorical pI-MW	Mascot search results		
					Anova (p)	Method B	Method A			Score	No. of matched peptides	Sequence coverage (%)
**2**	Myozenin-2	MYOZ2_MOUSE	Myoz2	Q9JJW5	0.00653522	0.0167378	**0.0506116**	3.02378	8.53-29800	130	9/11	30
**3**	Glutathione S-transferase P 1	GSTP1_MOUSE	Gstp1	P19157	0.0347651	**0.0616601**	0.0202928	3.03853	7.68- 23765	73	4/6	29
**4**	Myosin-1	MYH1_MOUSE	Myh1	Q5SX40	0.0238284	0.00220626	**0.00695099**	3.15057	5.60-224116	104	15/22	8
**5**	Actin, alpha skeletal muscle	ACTS_MOUSE	Acta1	P68134	0.04137	0.0481574	**0.153175**	3.18072	5.23-42366	191	11/12	45
**6**	Albumin	ALBU_MOUSE	Alb	P07724	0.00228815	0.0498459	**0.159345**	3.19675	5.75-70700	176	12/14	25
**7**	Glyceraldehyde-3-phosphate dehydrogenase	G3P_MOUSE	Gapdh	P16858	0.0250539	**0.068993**	0.0215258	3.20514	8.44- 36072	90	6/11	29
**9**	Adenylate kinase isoenzyme 1	KAD1_MOUSE	Ak1	Q9R0Y5	4.99×10^{-4}	0.0319092	**0.103079**	3.23039	5.67-21640	128	9/11	46
**10**	Superoxide dismutase [Mn], mitochondrial	SODM_MOUSE	Sod2	P09671	0.00408403	**0.0194791**	0.00579448	3.36166	8.80-24816	81	5/9	28
**11**	Dihydrolipoyllysine-residue acetyltransferase component of pyruvate dehydrogenase complex, mitochondrial **Fragment C-term**	ODP2_MOUSE	Dlat	Q8BMF4	0.0235998	**0.0119908**	0.00354491	3.38253	8.81-68469	84	6/8	12
**13**	Ankyrin repeat domain-containing protein 2	ANKR2_MOUSE	Ankrd2	Q9WV06	0.0159894	0.0105802	**0.0367369**	3.47224	5.95-36855	98	6/9	27
**14**	Malate dehydrogenase, cytoplasmic	MDHC_MOUSE	Mdh1	P14152	2.64×10^{-4}	0.0255662	**0.0898258**	3.51346	6.16-36659	139	8/8	30
**15**	UTP--glucose-1-phosphate uridylyltransferase	UGPA_MOUSE	Ugp2	Q91ZJ5	0.0261443	0.040836	**0.14366**	3.51796	7.18-57115	72	5/7	13
**16**	Fructose-bisphosphate aldolase A	ALDOA_MOUSE	Aldoa	P05064	0.00214931	**0.024366**	0.00685137	3.55638	8.31-39787	137	8/8	31
**17**	Beta-enolase	ENOB_MOUSE	Eno3	P21550	0.0147162	**0.0321504**	0.00903441	3.55866	6.73-47337	114	8/10	25
**19**	L-lactate dehydrogenase A chain **Fragment C-term**	LDHA_MOUSE	Ldha	P06151	0.00298447	**0.0276376**	0.00758	3.64612	7.62-36817	107	6/7	28
**20**	Telethonin	TELT_MOUSE	Tcap	O70548	0.00383146	0.00493785	**0.0180851**	3.66254	5.73-19294	139	7/8	46
**21**	Heat shock protein beta-6	HSPB6_MOUSE	Hspb6	Q5EBG6	3.58×10^{-4}	**0.217088**	0.0590855	3.67413	5.64-17567	91	5/9	46
**22**	Creatine kinase M-type	KCRM_MOUSE	Ckm	P07310	1.29×10^{-4}	0.0158004	**0.0583225**	3.69121	6.58-43246	208	14/18	37
**23**	Aldo-keto reductase family 1 member B1	ALDR_MOUSE	Akr1b1	P45376	9.12×10^{-4}	0.0217994	**0.0818126**	3.75297	6.71-36052	94	710	25
**24**	MIX									189		
	Troponin T, slow skeletal muscle	TNNT1_MOUSE	Tnnt1	O88346	0.00972135	0.0336224	**0.126473**	3.76157	5.26- 32221	147	13/25	39
	Isocitrate dehydrogenase [NAD] subunit alpha, mitochondrial	IDH3A_MOUSE	Idh3a	Q9D6R2					6.27-40069	69	7/25	28
**25**	Troponin T, fast skeletal muscle	TNNT3_MOUSE	Tnnt3	Q9QZ47	2.39×10^{-4}	0.0413539	**0.155843**	3.76853	5.26- 32221	121	11/19	34
**26**	Carbonic anhydrase 3	CAH3_MOUSE	Ca3	P16015	0.00369536	**0.11057**	0.0286215	3.86319	6.89-29633	188	11/15	59
**27**	Peptidyl-prolyl cis-trans isomerase A	PPIA_MOUSE	Ppia	P17742	8.54×10^{-4}	**0.0274004**	0.00704662	3.88844	7.74- 18131	73	4/6	28

**28**	Myosin regulatory light chain 11	MYL11_MOUSE	Myl11	P97457	0.0452157	**0.0637316**	0.0158068	4.03192	4.82- 19057	119	9/15	46
**29**	Myozenin-1	MYOZ1_MOUSE	Myoz1	Q9JK37	0.000068051	0.0336224	**0.126473**	3.76157	8.57-31438	142	9/10	38
**30**	Troponin T, slow skeletal muscle	TNNT1_MOUSE	Tnnt1	O88346	4.16 x10^{-4}	0.0497287	**0.202876**	4.07966	5.74-31325	149	9/13	35
**31**	Myozenin-1	MYOZ1_MOUSE	Myoz1	Q9JK37	0.00619141	**0.0403203**	0.00976304	4.12989	8.57-31438	74	6/10	23
**32**	NADH dehydrogenase [ubiquinone] flavoprotein 1, mitochondrial **Fragment C-term**	NDUV1_MOUSE	Ndufv1	Q91YT0	0.0219116	**0.0241047**	0.0057931	4.16093	8.51-51486	152	10/13	30
**33**	Myosin-1	MYH1_MOUSE	Myh1	Q5SX40	0.00404761	0.00562146	**0.0234575**	4.17285	5.60-224116	65	8/11	6
**35**	Myosin-1	MYH1_MOUSE	Myh1	Q5SX40	0.0429403	0.00554752	**0.0232714**	4.19491	5.60-224116	64	10/15	7
**36**	Myozenin-2	MYOZ2_MOUSE	Myoz2	Q9JJW5	1.65 x10^{-4}	0.0123619	**0.0529451**	4.28292	8.53-29800	95	6/8	20
**37**	Fructose-bisphosphate aldolase	ALDOA_MOUSE	Aldoa	P05064	0.00264145	**0.0671157**	0.0156579	4.28637	8.31- 39787	214	17/26	48
**38**	MIX									199		
	Cofilin-2	COF2_MOUSE	Cfl2	P45591	0.00316785	0.00316785	**0.00828273**	4.34314	7.66-18812	95	8/29	61
	Nucleoside diphosphate kinase B	NDKB_MOUSE	Nme2	Q01768					6.97-7466	129	12/29	63
**39**	Malate dehydrogenase, cytoplasmic **Fragment C-term**	MDHC_MOUSE	Mdh1	P14152	3.95 x10^{-4}	**0.102946**	0.00449586	22.8979	6.16-36659	110	8/13	28
**40**	Peroxiredoxin-1	PRDX1_MOUSE	Prdx1	P35700	7.30 x10^{-4}	**0.0814671**	0.0182386	4.46673	8.26-22390	85	5/8	30
**41**	Desmin	DESM_MOUSE	Des	P31001	0.0397346	**0.0520241**	0.0115213	4.51548	5.21-53522	118	8/9	20
**42**	ATP synthase subunit beta. mitochondrial	ATPB_MOUSE	Atp5f1b	P56480	0.0181938	**0.0329323**	0.00717896	4.58733	5.19-56265	134	8/8	20
**45**	Desmin	DESM_MOUSE	Des	P31001	0.0199237	**0.0620896**	0.0133068	4.66601	5.21-53522	175	12/13	26
**47**	Heat shock 70 kDa protein 1A	HS71A_MOUSE	Hspa1a	Q61696	0.00201321	**0.0319181**	0.00681551	4.68316	5.53-70321	91	8/12	13
**48**	Actin, alpha skeletal muscle	ACTS_MOUSE	Acta1	P68134	3.64 x10^{-4}	**0.134919**	0.028709	4.69952	5.23-42366	147	9/11	35
**49**	Myozenin-2	MYOZ2_MOUSE	Myoz2	Q9JJW5	1.47 x10^{-5}	0.00726689	**0.0341656**	4.70154	8.53-29800	165	11/14	40
**50**	Troponin T, slow skeletal muscle	TNNT1_MOUSE	Tnnt1	O88346	3.67 x10^{-4}	0.0248	**0.117886**	4.75346	5.74-31325	166	13/21	42
**52**	NADH dehydrogenase [ubiquinone] 1 alpha subcomplex subunit 8	NDUA8_MOUSE	Ndufa8	Q9DCJ5	0.00100857	**0.0519168**	0.0105206	4.93478	8.76-20435	72	4/6	25
**54**	Fructose-bisphosphate aldolase A	ALDOA_MOUSE	Aldoa	P05064	0.00508908	**0.0544885**	0.0110228	4.94323	8.31-39787	92	7/10	20
**55**	Troponin T, slow skeletal muscle	TNNT1_MOUSE	Tnnt1	O88346	2.46 x10^{-4}	0.0379486	**0.187948**	4.95269	5.74-31325	175	12/14	35
**56**	Trifunctional enzyme subunit alpha, mitochondrial **Fragment C-term**	ECHA_MOUSE	Hadha	Q8BMS1	5.43 x10^{-4}	**0.0319408**	0.0064318	4.96607	9.24-83302	89	7/9	12
**57**	Dihydrolipoyllysine-residue succinyltransferase component of 2-oxoglutarate dehydrogenase complex, mitochondrial **Fragment-C term**	ODO2_MOUSE	Dlst	Q9D2G2	3.19 x10^{-4}	**0.0479297**	0.00956407	5.01143	9.11-49306	135	10/11	19
**58**	Trifunctional enzyme subunit alpha, mitochondrial **Fragment N-term**	ECHA_MOUSE	Hadha	Q8BMS1	0.00822361	**0.0346225**	0.00690854	5.01156	9.24-83302	61	7/11	12
**59**	Myozenin-2	MYOZ2_MOUSE	Myoz2	Q9JJW5	0.00116498	**0.0389552**	0.00773393	5.03692	8.53-29800	151	10/12	39
**60**	UTP--glucose-1-phosphate uridylyltransferase	UGPA_MOUSE	Ugp2	Q91ZJ5	0.0102323	0.0112605	**0.0577956**	5.13258	7.18-57115	61	5/10	13
**61**	Myosin light chain 3	MYL3_MOUSE	Myl3	P09542	0.0344769	**0.0285028**	0.00544332	5.23628	5.03-22521	193	10/11	59
**62**	Albumin	ALBU_MOUSE	Alb	P07724	0.023357	**0.0354937**	0.0066367	5.34809	5.81-73701	154	10/11	20
**63**	Aconitate hydratase, mitochondrial	ACON_MOUSE	Aco2	Q99KI0	0.00373193	**0.0628969**	0.0114695	5.48382	8.08-86151	161	12/15	24
**64**	Actin, alpha skeletal muscle	ACTS_MOUSE	Acta1	P68134	0.00199016	**0.0591132**	0.0107746	5.48633	5.23-42366	90	5/5	17
**65**	Glyceraldehyde-3-phosphate dehydrogenase	G3P_MOUSE	Gapdh	P16858	0.0275617	**0.0384902**	0.00695229	5.53634	8.44- 36072	66	5/7	20
**67**	Troponin I, fast skeletal muscle	TNNI2_MOUSE	Tnni2	P13412	6.81 x10^{-4}	**0.136028**	0.0236175	5.75961	8.65-21515	84	8/12	24

**68**	Beta-enolase	ENOB_MOUSE	Eno3	P21550	9.83 x10^{-4}	**0.0568846**	0.00975399	5.83194	6.73-47337	190	11/12	37
**69**	Pyruvate kinase PKM	KPYM_MOUSE	Pkm	P52480	0.0158384	**0.0178872**	0.00306301	5.83975	7.18-58378	92	8/12	19
**71**	Phosphoglycerate kinase 1	PGK1_MOUSE	Pgk1	P09411	0.00804836	**0.0535783**	0.00905801	5.91501	8.02-44921	77	6/8	22
**72**	Myosin regulatory light chain 2, ventricular/cardiac muscle isoform	MLRV_MOUSE	Myl2	P51667	5.85 x10^{-4}	**0.167455**	0.0279587	5.98939	4.86-18852	197	13/15	68
**73**	Trifunctional enzyme subunit alpha, mitochondrial **Fragment C-term**	ECHA_MOUSE	Hadha	Q8BMS1	8.24 x10^{-4}	**0.0981757**	0.0162945	6.0251	9.24-83302	104	9/10	11
**74**	Triosephosphate isomerase	TPIS_MOUSE	Tpi1	P17751	2.97 x10^{-4}	**0.214687**	0.0355957	6.03126	6.90-27038	277	13/17	67
**75**	Myosin light chain 3	MYL3_MOUSE	Myl3	P09542	0.00911065	**0.0769502**	0.012673	6.07196	5.03-22521	147	8/10	45
**76**	Glyceraldehyde-3-phosphate dehydrogenase	G3P_MOUSE	Gapdh	P16858	0.00205939	0.00823743	**0.0512264**	6.21874	8.44- 36072	68	7/11	20
**77**	Beta-enolase	ENOB_MOUSE	Eno3	P21550	9.83 x10^{-4}	**0.0231914**	0.00371458	6.24335	6.73-47337	159	10/13	35
**78**	ATP synthase subunit beta, mitochondrial	ATPB_MOUSE	Atp5f1b	P56480	9.48 x10^{-4}	**0.0249091**	0.00398799	6.24604	5.19-56265	109	10/18	22
**79**	Actin, alpha skeletal muscle	ACTS_MOUSE	Acta1	P68134	1.43 x10^{-4}	**0.243898**	0.038305	6.36727	5.23-42366	113	7/9	28
**80**	Troponin T, fast skeletal muscle **Fragment N-term**	TNNT3_MOUSE	Tnnt3	Q9QZ47	0.00426799	**0.148746**	0.0229017	6.495	5.26- 32221	98	9/13	19
**82**	Phosphoglycerate mutase 2	PGAM2_MOUSE	Pgam2	O70250	0.00247434	**0.141089**	0.0204806	6.8889	8.65-28980	154	9/10	42
**83**	Myosin-7 **Fragment C-term**	MYH7_MOUSE	Myh7	Q91Z83	0.0315985	0.00157128	**0.0108882**	6.92949	5.59- 223539	63	12/22	7
**84**	Adenylate kinase isoenzyme 1	KAD1_MOUSE	Ak1	Q9R0Y5	0.00155791	0.0194984	**0.137701**	7.06214	5.67-21640	93	5/6	37
**86**	Electron transfer flavoprotein subunit beta	ETFB_MOUSE	Etfb	Q9DCW4	0.0355916	**0.0538277**	0.00757016	7.11051	8.24- 27834	88	5/6	28
**88**	Beta-enolase	ENOB_MOUSE	Eno3	P21550	0.00385019	**0.0856925**	0.0115423	7.4242	6.73-47337	195	14/16	33
**91**	Myosin light chain 3	MYL3_MOUSE	Myl3	P09542	0.00504101	**0.0380408**	0.00475411	8.00167	5.03-22521	165	10/12	50
**92**	Myosin-1 **Fragment C-term**	MYH1_MOUSE	Myh1	Q5SX40	0.023574	0.00209212	**0.0169456**	8.09974	5.60-224116	71	9/12	6
**93**	Actin, alpha skeletal muscle **Fragment N-term**	ACTS_MOUSE	Acta1	P68134	0.00480125	**0.0626546**	0.00764117	8.1996	5.23-42366	76	5/7	15
**94**	Albumin **Fragment N-term**	ALBU_MOUSE	Alb	P07724	0.0206167	**0.020242**	0.00246353	8.21665	5.75-70700	144	9/9	19
**95**	Phosphoglycerate kinase 1	PGK1_MOUSE	Pgk1	P09411	0.0650786	**0.0420672**	0.00508995	8.26476	8.02-44921	73	6/9	22
**97**	Troponin T, fast skeletal muscle **Fragment N-term**	TNNT3_MOUSE	Tnnt3	Q9QZ47	5.94 x10^{-5}	**0.161313**	0.0189587	8.50869	5.74-31325	59	6/9	13
**98**	NADH dehydrogenase [ubiquinone] 1 beta subcomplex subunit 10	NDUBA_MOUSE	Ndufb10	Q9DCS9	1.43 x10^{-4}	0.0298348	**0.262432**	8.79618	8.19-21296	137	8/11	48
**99**	Troponin T, fast skeletal muscle	TNNT3_MOUSE	Tnnt3	Q9QZ47	0.00264028	0.00463017	**0.040861**	8.82495	5.26- 32221	137	11/15	34
**100**	Troponin T, slow skeletal muscle	TNNT1_MOUSE	Tnnt1	O88346	3.84 x10^{-4}	0.0067815	**0.0598502**	8.82551	5.74-31325	145	9/13	35
**101**	Actin, alpha skeletal muscle	ACTS_MOUSE	Acta1	P68134	6.23 x10^{-7}	**0.283608**	0.03213	8.8269	5.23-42366	104	6/7	28
**102**	Troponin T, fast skeletal muscle **Fragment N-term**	TNNT3_MOUSE	Tnnt3	Q9QZ47	4.88 x10^{-6}	**0.143221**	0.0158016	9.0637	5.26-32221	63	5/7	12
**104**	ATP synthase subunit alpha, mitochondrial **Fragment N-term**	ATPA_MOUSE	Atp5f1a	Q03265	6.22 x10^{-4}	**0.109444**	0.0114835	9.53051	9.22-59830	163	15/18	28
**105**	Fructose-bisphosphate aldolase	ALDOA_MOUSE	Aldoa	P05064	0.00277212	**0.122317**	0.0128274	9.53564	8.31- 39787	224	17/21	45
**106**	Glyceraldehyde-3-phosphate dehydrogenase	G3P_MOUSE	Gapdh	P16858	0.00832791	**0.082468**	0.0084289	9.78396	8.44-36072	61	4/7	14
**107**	Fructose-bisphosphate aldolase	ALDOA_MOUSE	Aldoa	P05064	7.60 x10^{-4}	**0.135807**	0.0136956	9.91611	8.31- 39787	198	12/13	45
**108**	Creatine kinase M-type	KCRM_MOUSE	Ckm	P07310	8.67 x10^{-5}	**0.0260892**	0.00256392	10.1755	6.58-43246	60	4/6	8
**110**	Heat shock cognate 71 kDa protein	HSP7C_MOUSE	Hspa8	P63017	1.52 x10^{-4}	**0.139983**	0.0132307	10.5802	5.37-71055	165	11/12	24
**113**	Trifunctional enzyme subunit alpha, mitochondrial **Fragment N-term**	ECHA_MOUSE	Hadha	Q8BMS1	5.87 x10^{-4}	**0.0702914**	0.00611436	11.4961	9.24-83302	133	13/14	20
**114**	ATP synthase subunit alpha, mitochondrial **Fragment C-term**	ATPA_MOUSE	Atp5f1a	Q03265	3.32 x10^{-4}	**0.104268**	0.00833941	12.5031	9.22-59830	114	9/12	22

**115**	Troponin T, fast skeletal muscle	TNNT3_MOUSE	Tnnt3	Q9QZ47	6.38 x10^{-5}	0.00581789	**0.0741273**	12.7413	5.26- 32221	142	13/21	38
**116**	ATP synthase subunit alpha, mitochondrial **Fragment C-term**	ATPA_MOUSE	Atp5f1a	Q03265	0.0115545	**0.0343452**	0.00255957	13.4184	9.22- 59830	74	6/8	15
**118**	Glyceraldehyde-3-phosphate dehydrogenase	G3P_MOUSE	Gapdh	P16858	1.26 x10^{-4}	**0.381609**	0.0280007	13.6285	8.44- 36072	210	15/19	51
**119**	Troponin T. fast skeletal muscle Fragment	TNNT3_MOUSE	Tnnt3	Q9QZ47	6.16 x10^{-4}	**0.200568**	0.0136115	14.7352	5.26-32221	80	8/13	15
**120**	ATP synthase subunit alpha, mitochondrial **Fragment C-term**	ATPA_MOUSE	Atp5f1a	Q03265	0.0838683	**0.0890906**	0.00594253	14.992	9.22-59830	90	8/13	18
**121**	Troponin T, slow skeletal muscle	TNNT1_MOUSE	Tnnt1	O88346	0.0232728	**0.0703075**	0.00465498	15.1037	5.74-31325	176	15/18	32
**122**	Malate dehydrogenase, cytoplasmic **Fragment C-term**	MDHC_MOUSE	Mdh1	P14152	9.37 x10^{-4}	**0.0372142**	0.002385	15.6034	6.16-36659	69	5/10	21
**123**	Actin, alpha skeletal muscle	ACTS_MOUSE	Acta1	P68134	0.00126686	**0.0958159**	0.0056055	17.0932	5.23-42366	103	6/7	25
**124**	Adenylate kinase isoenzyme 1	KAD1_MOUSE	Ak1	Q9R0Y5	3.57 x10^{-5}	**0.144059**	0.00838602	17.1784	5.67-21640	96	8/13	46
**126**	ATP synthase subunit alpha, mitochondrial **Fragment C-term**	ATPA_MOUSE	Atp5f1a	Q03265	2.32 x10^{-4}	**0.0650761**	0.00364188	17.8688	9.22-59830	74	7/13	17
**127**	Troponin T, fast skeletal muscle	TNNT3_MOUSE	Tnnt3	Q9QZ47	5.94 x10^{-5}	0.00493462	**0.0895241**	18.142	5.26- 32221	113	11/22	34
**128**	Aconitate hydratase, mitochondrial	ACON_MOUSE	Aco2	Q99KI0	0.00838893	**0.0643681**	0.00335543	19.1832	8.08-86151	140	9/9	17
**131**	Adenylate kinase isoenzyme 1	KAD1_MOUSE	Ak1	Q9R0Y5	6.37 x10^{-4}	**0.0544682**	0.00255464	21.3212	5.67-21640	147	11/16	55
